# Operational failures and how they influence the work of GPs: a qualitative study in primary care

**DOI:** 10.3399/bjgp20X713009

**Published:** 2020-09-22

**Authors:** Carol Sinnott, Alexandros Georgiadis, Mary Dixon-Woods

**Affiliations:** THIS Institute, University of Cambridge, Cambridge;; ICON plc, the Translation and Innovation Hub Building, Imperial College London, London.; THIS Institute, University of Cambridge, Cambridge;

**Keywords:** general practice, organisational efficiency, primary health care, psychological burnout, task performance and analysis, workflow

## Abstract

**Background:**

Operational failures, defined as inadequacies or errors in the information, supplies, or equipment needed for patient care, are known to be highly consequential in hospital environments. Despite their likely relevance for GPs’ experiences of work, they remain under-explored in primary care.

**Aim:**

To identify operational failures in the primary care work environment and to examine how they influence GPs’ work.

**Design and setting:**

Qualitative interview study in the East of England.

**Method:**

Semi-structured interviews were conducted with GPs (*n* = 21). Data analysis was based on the constant comparison method.

**Results:**

GPs reported a large burden of operational failures, many of them related to information transfer with external healthcare providers, practice technology, and organisation of work within practices. Faced with operational failures, GPs undertook ‘compensatory labour’ to fulfil their duties of coordinating and safeguarding patients’ care. Dealing with operational failures imposed significant additional strain in the context of already stretched daily schedules, but this work remained largely invisible. In part, this was because GPs acted to fix problems in the here-and-now rather than referring them to source, and they characteristically did not report operational failures at system level. They also identified challenges in making process improvements at practice level, including medicolegal uncertainties about delegation.

**Conclusion:**

Operational failures in primary care matter for GPs and their experience of work. Compensatory labour is burdensome with an unintended consequence of rendering these failures largely invisible. Recognition of the significance of operational failures should stimulate efforts to make the primary care work environment more attractive.

## INTRODUCTION

Though primary care is essential to equitable, high-quality, and cost-effective health services,^1^ its sustainability is increasingly challenged by both the number of GPs leaving the profession and problems in recruitment.^2^^,^^3^ Two-thirds of GPs report unmanageable workloads,^4^ and almost half report emotional exhaustion associated with their work.^5^ These pressures have been variously attributed to substantial increases in consultation rates, the complexity of patients’ needs, and growing policy expectations to move more care from hospitals to the community.^4^^,^^6^^,^^7^ Efforts to address the workforce crisis, including enhanced recruitment and retention strategies and diversification of skill-mix in general practice, have been welcomed.^8^ However, there has been noticeably less focus on the work environments of GPs,^3^^,^^7^ even though the conditions in which people work, including the extent to which operational processes are supportive of the goals of work, are known to be highly consequential for worker satisfaction.^9^^,^^10^

Research in hospitals has clearly identified the impact of suboptimal work conditions on staff efficiency and morale. Operational failures — defined as disruptions, errors, or inadequacies in the information, supplies, or equipment needed for patient care^11^ — occur frequently in secondary care settings. These failures frustrate employees, decrease individual and organisational performance, and increase risks to patients.^11^^–^^13^ Often, they may appear initially as small problems (for example, thermometer probe covers going missing or incorrect medications delivered to wards), but cumulatively can be highly impactful because they push healthcare professionals to use workarounds or time-consuming adjustments to get tasks done.^12^ Hospital-based research shows that 9% of a healthcare professional’s working day can be spent dealing with operational failures,^11^ but once recognised they are tractable to improvement through systems redesign with attendant benefits in organisational efficiency and job satisfaction.^14^

The authors recently reviewed the international medical literature on operational failures in primary care,^15^ and found that it has remained an under-researched area. In this study, qualitative methods were used to identify the operational failures reported in everyday practice by NHS GPs, and how they influence GPs’ work and experiences of work was explored.

## METHOD

### Design

A qualitative interview study was conducted with GPs. ‘Operational failures’ were used as a sensitising concept^16^ to facilitate exploration of problems in primary care work systems. Primary care work systems were defined as anyone or anything the GP must interact with in order to provide care.^17^

**Table 1:** How this fits in

Operational failures, defined as inadequacies or errors in the information, supplies, or equipment needed for patient care, are known to be highly consequential in hospital environments. This qualitative study shows that operational failures are also common and burdensome in UK primary care. Examples included problems in the supply of information to GPs from external healthcare providers, technology problems, and missing or broken equipment. These problems required what was termed ‘compensatory labour’ to address them. Although GPs’ compensatory labour usually resolved the problem more quickly in the short term than did redirecting failures to their source, it may in fact be counterproductive in the longer term by rendering invisible at system level the operational failures themselves and the possible improvement opportunities associated with them.

### Sampling

GPs were invited to participate via a regional Clinical Research Network,^18^ using criteria relating to:

- length of time qualified (≥/<10 years);
- location (rural/urban); and
- practice size (≤5 GPs/>5 GPs).

The sample size was not defined in advance but the authors continued to interview GPs until they were confident that data saturation had been reached, which was determined when additional data no longer added to the advancement of the analysis.^16^

### Data collection

Interviews were conducted by an academic GP and a health services researcher, both experienced qualitative researchers. Interviews took place in participants’ practices or by telephone between February and July 2018. The interview topic guide was informed by a recent critical interpretive synthesis on operational failures in primary care^15^ (see Supplementary Box S1 for details). As well as general questions, GPs were asked to describe operational failures they had experienced in their most recent clinical session, using their own schedules and patient notes as an aide-memoire, a technique known as chart-stimulated recall.^19^ The topic guide was modified iteratively to pursue emergent themes.

### Analysis

All interviews were audio-recorded, transcribed in full, and anonymised. Analysis was based on the constant comparative method.^16^ All interviews were read and coded independently by the interviewers. Initially, types of operational failure were open coded, as were the conditions, consequences, and actions associated with these failures. In the second stage of coding, related codes were drawn together and the data synthesised using sensitising constructs from systems engineering^20^ and Hollnagel’s Safety-II concepts^21^ (see Supplementary Box S2 for details). Field notes and memos were discussed during team meetings to facilitate theoretical development. Quotations were selected by consensus to most closely reflect typical responses and diversity within codes. NVivo (version 12) was used to facilitate data management. Assurances of confidentiality and anonymity of interview data were provided to participants, and written informed consent was obtained to digitally record the interviews.

## RESULTS

Twenty-three GPs working in 16 practices responded positively to the invitation to participate and, of these, 21 were interviewed. Eleven worked in urban practices, 14 had been practising as GPs for ≥10 years, and 14 worked in practices of ≥5 GPs. Median interview duration was 29 minutes (range = 12–48 minutes). It was found that GPs reported multiple operational failures that were hugely burdensome, and required what was termed ‘compensatory labour’ to address them.

### Operational failures encountered during GPs’ work

GPs reported a significant burden of operational failures. The most common failures related to problems in the supply of information to them from sources outside of their own practice. Delayed or missing hospital discharge letters were frequent, but excessive information from hospitals was reported to be as much of a problem as too little information. For instance, GPs described important information being lost in 12-page discharge summaries where they *‘couldn’t work out the wood from the trees’* (GP18). Hospital letters often included recommendations for specific investigations or medications for patients but left it to GPs to clarify who was responsible for implementing them. Efforts to get necessary information from hospitals were further frustrated by hospital protocols stating that certain information (for example, laboratory results) could not be shared with practice clerical staff:

‘Time is often taken up with the interface for communication between us and secondary care. So letters that were meant to come but didn’t come, letters are not giving enough information, or not explicit. This morning I had a letter that was pretty ropey in all respects … if they’d just given me the dose it would have been straightforward .’

(GP2)

‘Information clutter is an absolutely overwhelmingly gargantuan issue.’

(GP6)

Operational failures also complicated GPs’ communication with hospitals, sometimes resulting in delays for patients. For instance, changes in care pathways and requirements for specific forms that themselves changed often made organising secondary care difficult and frustrating, with GPs’ referrals sometimes returned to the practice without any clinical action being taken:

‘We used to refer to the clinician that we thought was the correct clinician to see the patient, now we don’t have that option, they have to go through a central service, which causes a delay for the patient — the patient gets frustrated, we end up having to see the patient more often, that causes more inefficiency for the GP.’

(GP19)

GPs also reported problems associated with electronic referral systems to community and social care, which had replaced previous direct contact with colleagues. Electronic referrals left GPs uncertain when the patient would be seen, hindered their ability to offer multidisciplinary community-based care, and pushed them to refer patients to hospital that would otherwise have been cared for at home:

*‘All of us can spend an hour sometimes trying to get somebody some care to keep them at home, and often there isn’t any, and we go round and round in circles. We spend an hour phoning up and then we get nowhere. So, I think we’ve all given up now. And then, of course, we send the people to hospital inappropriately* […]*but we feel guilty because it’s just not how we’re meant to practise.’*

(GP18)

Communication with community pharmacies was also vulnerable to inadequate information transfer, for example, through the loss of electronic prescriptions in the journey between practice and pharmacy. This is an issue that was often left to GPs to resolve:

*‘People say I ordered my prescription three days ago, for some reason they haven’t got the prescription between the prescription system or the pharmacist* […]*so you find the receptionist coming into the room to say we can’t find this prescription, it’s lost, can you duplicate, print and sign it.’*

(GP11)

Processes within practices themselves were not immune to operational failures. GPs reported that their work was disrupted by failures relating to equipment, including broken electrocardiograph machines, missing thermometers, and unstocked materials such as urine containers. Technology problems, such as crashing or non-booting computers, interfered frequently with access to electronic health records and added pressure to already strained 10-minute consultations:

‘I was trying to do a prescription for a patient. The computer froze. I couldn’t get it to unfreeze. I stopped that consultation, had to re-boot the computer and then go back into all the different systems that I was in, get the prescription out and the patient had to wait ten minutes, it put me behind; that often happens.’

(GP1)

GPs also reported direct interruptions of their work by other practice staff. Some practices had introduced a system where all urgent queries were directed to the ‘duty doctor’ (a GP rostered to triage phone calls and see emergency cases) with the intention of shielding remaining GPs from unpredictable interruptions and ensuring their clinical sessions ran smoothly. Not all practices used this approach, and where it was in place it was often stressful for the doctor involved:

*‘This is why we’ve brought the duty doctor system, so that all those interruptions go to somebody who is not running a normal surgery* […] *I agree we need to get to the bottom of the problem, but we feel that a typical day should be as smooth as possible.’*

(GP11)

‘*On a duty doctor day you will be disturbed significantly in terms of calls, knocks on the door, facilitating the nursing staff if they’ve got any questions plus dealing with any queries from reception.*’

(GP5)

GPs in rural practices reported spending more time travelling to home visits, so failures relating to home visits disproportionately affected their work. Examples included being called out for duties that other services (for example, district nurses, community emergency response team) did not have the capacity to deliver, wasted journeys due to incorrect patient addresses, or being called to write up drug charts for care home patients that could have been written by hospital doctors:

*‘We had a patient who had just been discharged from hospital to a nursing home* […] *they* [the hospital staff] *hadn’t written the drug chart. So this came in to be done urgently. And the system failure on our part was that the administrative staff who took the call to book the home visit did not put the new address* […] *so I went to their home and they were not there.’*

(GP11)

### Compensatory labour is required to address operational failures

GPs reported that, as patients’ first point of contact with the health service and as the physician responsible for generalist longitudinal care, they were exposed to operational failures in all facets of the healthcare system. GPs felt that their secondary and community care colleagues had both *‘unrealistic expectations’* (GP20) of what general practice could deliver and an inaccurate view of GPs as the *‘default person to look after things’* (GP8). These expectations compounded the effects of operational failures in primary care, resulting in GPs doing work that they felt would be done more quickly or effectively by others, was the clear responsibility of others, or for which they lacked system-level supports:

‘As a GP, I liaise with community rehabilitation or nursing or palliative care or other services and because these various services are quite fragmented and difficult to communicate with, certainly in the last year or two, that interferes or interrupts those kind of tasks. Coordinating complicated care often takes a lot longer than it used to.’

(GP14)

‘They make it very clear that they expect you to do it, and it’s really not appropriate — that can be annoying.’

(GP4)

GPs felt that, as the presumed coordinator of their patients’ care, there was an onus on them to work around the operational failures they encountered. As a result, they were forced into the role of compensating for operational failures, a role that had significant impacts on the character and volume of their work:

‘I’m in the boat where I’d rather do the best for my patient, so if it’s quicker for me to do it, I will just do it, I will take that extra work.’

(GP17)

‘If you’re busy and there’s resistance, you end up just caving in and getting on with it because it’ll be quicker that way.’

(GP1)

The bridging actions taken by GPs to close the gap between what patients needed and the operational failures that got in the way of meeting those needs were labelled as compensatory labour. Compensatory labour was required by the ubiquity of operational failures combined with GPs’ deeply felt responsibilities for synthesising information and coordinating care. The tasks of compensatory labour were characteristically mundane, but, in the context of highly pressurised schedules, they imposed significant burdens on GPs; the frequency of individually small compensations meant that cumulative time losses were highly impactful. Each extra step a GP had to take to deal with an operational failure added to the complexity of completing a task. Having to undertake often very significant amounts of compensatory labour actively configured the work that GPs were doing on a daily basis:

‘… you end up having to write a lot of letters to chase things up — the patient has already been to see you once, you then have to contact them again to explain the results. So, you end up doing three steps, where there could have been just one.’

(GP19)

*‘I’m currently waiting on a clinic letter from a consultant that’s been at least around a month* […] *I will phone the consultant directly and the liaison officer can also help … you make the best of it, you just have to get around the problems.’*

(GP12)

Compensatory labour also required trade-offs. Addressing failures as they occurred might achieve short-term benefits for one patient, but shifted risk to other patients by virtue of time pressures or cognitive overload:

*‘It’s extra time for us to look through the notes, to chase up on blood test results and sort the problems out* […] *that limits the time for us doing other things.’*

(GP15)

Repeatedly compensating for operational failures that were not of their own doing and were not related to their professional training added to GPs’ feelings of stress and low morale:

‘You feel quite stressed — I like things working efficiently instead of adding to your workload.’

(GP13)

Despite the burdensome nature of compensatory labour, GPs’ actions to remedy failures were generally invisible. Perhaps because compensating for the problems usually resolved them more quickly in the short term than redirecting them to the source, or perhaps because reporting operational failures was perceived as futile, only a small minority reported system-level operational failures to authorities such as GP liaison officers, commissioners, and others:

*‘I know that I’ve got to write two letters today to the GP liaison officers — it just takes time* […] *The frustrating thing with the hospital is you write to them in the hope that they can learn from it and improve it, but what they do is they just fob you off.’*

(GP20)

A further reason not to formally report operational failures lay in the perception that the patient safety threats they posed were not as great as in secondary care, or that they could be more readily addressed by compensatory labour. In contrast, participants did describe regular meetings (for example, quarterly) to discuss significant safety events:

‘In a hospital environment, if the equipment is not working and someone is being brought in by ambulance with an acute coronary syndrome and that equipment … you can’t say come back on Monday because that patient might be dead. So in general practice it’s inconveniences and delays but very rarely harm.’

(GP14)

*‘… a significant event — usually it’s big, some harm has to come to the patient. But I really think we haven’t really focused on these nitty-gritty* [operational failure] *issues.’*

(GP11)

GPs were also often reluctant to attempt to change processes within their own practices, in part because they felt they lacked time or capacity to design, implement, quality assure, and oversee new processes. A prominent feature of interviews was that GPs reported simply trying to get through the pressures of their work each day rather than make proactive operational changes, describing their current situation as *‘running bloody fast on the treadmill’* (GP6). Operational systems in smaller practices appeared to benefit from greater continuity with patients, but these practices struggled in other ways, such as generating the slack internally to reorganise practice processes:

‘… if you can achieve better continuity of care with the patient, so you know the patient, and you know a bit more about them, that does streamline the system better.’

(GP19)

*‘Each change needs space to breathe and the capacity to do it and actually there’s a question of safety, while you are experimenting with this, while you’ve got* [administrative staff] *attempting to start coding your records, you need to create GP capacity to cross-check the coding, and actually when we are all at breaking point it is quicker to do it myself.’*

(GP9)

A second challenge to process redesign was that the obvious solution for many issues — delegating to other practice staff — was a source of anxiety for GPs. They felt that delegation could further complicate workflows, increase the risk of something being missed, or might not be acceptable from a medicolegal perspective:

‘When these failures arise, it’s just easier for me to do them, as opposed to try and find a way to delegate them.’

(GP10)

*‘It’s interesting the level of responsibility that the GMC* [General Medical Council] *and the legal side of things put on GPs. There’s a general confusion in primary care about what can be delegated and what can’t and how you can delegate and create at our scale teams with enough resilience to actually be able to cope with pathways of work, making the rules clear that this is appropriate for somebody with no clinical expertise, this is your role, this is your set. So, we do vast quantities of administrative work that is soul-destroying.’*

(GP9)

## DISCUSSION

### Summary

This study has identified the nature of operational failures that confront GPs, and suggests that these failures are profoundly consequential for their experiences of work. Poorly designed and suboptimal work systems that GPs in this study described were indicative of a yawning gap between ‘work-as-imagined’ (an idealised view of work tasks that disregards how task performance must be adjusted to match the constantly changing conditions in the work environment) and ‘work-as-done’ (what actually happens as work unfolds in time in complex contexts).^13^^,^^21^ GPs’ actions to plug this gap required substantial compensatory labour of an especially enervating kind: it made poor use of their knowledge and skills, and consumed scarce resources of time and energy.^22^ Many (though not all) sources of operational failures were located outside of practices themselves, but mechanisms for reporting external failures were used rarely, not least because GPs were likely to try to fix a problem in the here-and-now in order to ensure patients were not disadvantaged rather than seek to raise the problem at system level. For problems arising internal to practices, GPs were deterred by the scale of the effort required and uncertainties about some of the medicolegal issues, indicating that high-quality multi-modal support may be needed to improve processes at practice level. Ironically, GPs’ compensatory labour may in fact be counterproductive in the longer term by rendering invisible at system level the operational failures themselves and the possible opportunities for improvement.^11^^,^^12^^,^^14^^,^^23^

### Comparison with existing literature

Recent studies on why GPs choose to leave clinical practice have identified reasons such as ‘surprise work’ (for example, the unpredictable extra demands on GPs’ time),^24^ diminished clarity around professional boundaries, poorly balanced work demands, and concerns about the scope, limits, and legal and professional liabilities associated with delegation.^3^^,^^4^^,^^25^^,^^26^ This study’s findings build on these studies, showing how GPs are forced into an additional invisible (and unremunerated) workload of compensatory labour in the face of operational failures that is likely to contribute to stress, burnout, and possibly exits from clinical practice.

Much of the previous research on operational failures has been conducted in secondary care environments. Studies on hospital-based operational failures^11^^,^^12^ have identified that they often concern defects in supply chains for equipment and other supplies. In contrast, this study found that primary care failures were more dominated by problems in communication of patient-related information or mismatches in role expectations and responsibilities. It further found, as has previously been reported in the secondary care literature, that failures in primary care were met with resilience in the form of workarounds, and the term ‘compensatory labour’ has been proposed to describe the work GPs do to fill the gap between what patients need and the operational failures that get in the way of addressing that need.

### Strengths and limitations

A bottom-up qualitative approach was necessary to generate new understandings on system-level failures in primary care, which had previously been under-explored. A limitation is that the sample involved only one region of England, but the transferability of findings is supported by the diversity of settings including inner-city and rural practices, large and small practices, and GP participants ranging from those recently qualified to those with significant practice experience. A strength of the study was its focus on examining work-as-done rather than the rarer (and potentially more discomfiting) topics of errors or critical incidents associated with operational failures. Although it is unlikely that the complete set of operational failures experienced by GPs was captured, eliciting the problems experienced in the most recent clinical session helped to ensure the data related credibly to routine care, and mitigated the risk of inaccurate recall. The multidisciplinary research team lent confirmability to the analysis by creating opportunities for reflexivity and demonstrating consistency during interviewing, coding, and analysis.

### Implications for research and practice

By identifying the operational failures that routinely affect GPs working in the NHS, this study has illustrated how work-asdone in general practice involves a largely invisible but highly consequential burden of compensatory labour. As others have also argued, in the context of an NHS Long Term Plan that gives primacy to the delivery of primary medical and community health services,^27^ commissioners and policymakers must resist the urge to place additional responsibilities on general practice until the gap between work-as-imagined and work-as-done is better understood.^7^ New initiatives such as Sustainability and Transformation Partnerships, Integrated Care Systems, and Primary Care Networks present opportunities for GPs and colleagues in secondary and community care to work together on improving systems for communicating with each other, but these findings signal that changes should be designed for the world that is inhabited by GPs rather than an imagined or idealised world. Urgent clarification is also needed on some medicolegal aspects of change. Further, new changes in the division of responsibilities between primary and secondary care should not leave GPs straining to meet heightened policy expectations within an otherwise unchanged health system. Finally, efforts to address operational failures will benefit from learning from what goes right: the operational successes. The literature on positive deviance has shown that the ability to solve a problem may already exist within the community experiencing the problem, and the challenge is to find and share particular practices or solutions already in use that may be of benefit to all.^28^ Mechanisms to facilitate identification, harmonisation, and implementation are likely to be of particular value.^29^

Operational failures are common in general practice, and force stretched GPs to take additional steps to get patient-related work done. These compensatory actions may be hidden from the view of commissioners, policymakers, and other healthcare professionals, but are an important threat to GPs’ job satisfaction, patient safety, and the quality of care. Research is now required to quantify the impact of different operational failures in terms of time consumed, GPs’ efficiency, and patient care. Efforts to link specific operational failures with serious downstream errors will further determine which failures to prioritise for improvement in an evidence-based way.^12^^,^^23^
